# A Basomedial Amygdala to Intercalated Cells Microcircuit Expressing PACAP and Its Receptor PAC1 Regulates Contextual Fear

**DOI:** 10.1523/JNEUROSCI.2564-20.2021

**Published:** 2021-04-14

**Authors:** Abha K. Rajbhandari, Christopher J. Octeau, Sarah Gonzalez, Zachary T. Pennington, Farzanna Mohamed, Jeremy Trott, Jasmine Chavez, Erin Ngyuen, Natasha Keces, Weizhe Z. Hong, Rachael L. Neve, James Waschek, Baljit S. Khakh, Michael S. Fanselow

**Affiliations:** ^1^Department of Psychology, University of California, Los Angeles, California 90095; ^2^Staglin Center for Brain and Behavior, University of California, Los Angeles, California 90095; ^3^Department of Physiology, University of California, Los Angeles, California 90095; ^4^Department of Neurobiology, University of California, Los Angeles, California 90095; ^5^Gene Delivery Technology Core, Massachusetts General Hospital, Boston, Massachusetts, California 02114; ^6^Department of Psychiatry, University of California, Los Angeles, California 90095; ^7^Department of Psychiatry, Icahn School of Medicine at Mount Sinai, New York, New York 10029

**Keywords:** amygdala, fear, PACAP

## Abstract

Trauma can cause dysfunctional fear regulation leading some people to develop disorders, such as post-traumatic stress disorder (PTSD). The amygdala regulates fear, whereas PACAP (pituitary adenylate activating peptide) and PAC1 receptors are linked to PTSD symptom severity at genetic/epigenetic levels, with a strong link in females with PTSD. We discovered a PACAPergic projection from the basomedial amygdala (BMA) to the medial intercalated cells (mICCs) in adult mice. *In vivo* optogenetic stimulation of this pathway increased CFOS expression in mICCs, decreased fear recall, and increased fear extinction. Selective deletion of PAC1 receptors from the mICCs in females reduced fear acquisition, but enhanced fear generalization and reduced fear extinction in males. Optogenetic stimulation of the BMA-mICC PACAPergic pathway produced EPSCs in mICC neurons, which were enhanced by the PAC1 receptor antagonist, PACAP 6-38. Our findings show that mICCs modulate contextual fear in a dynamic and sex-dependent manner via a microcircuit containing the BMA and mICCs, and in a manner that was dependent on behavioral state.

**SIGNIFICANCE STATEMENT** Traumatic stress can affect different aspects of fear behaviors, including fear learning, generalization of learned fear to novel contexts, how the fear of the original context is recalled, and how fear is reduced over time. While the amygdala has been studied for its role in regulation of different aspects of fear, the molecular circuitry of this structure is quite complex. In addition, aspects of fear can be modulated differently in males and females. Our findings show that a specific circuitry containing the neuropeptide PACAP and its receptor, PAC1, regulates various aspects of fear, including acquisition, generalization, recall, and extinction in a sexually dimorphic manner, characterizing a novel pathway that modulates traumatic fear.

## Abstract (precis)

Trauma can cause dysfunctional fear regulation leading some people to develop disorders, such as post-traumatic stress disorder (PTSD). The amygdala regulates fear, whereas PACAP (pituitary adenylate activating peptide) and PAC1 receptors are linked to PTSD symptom severity at genetic/epigenetic levels, with a strong link in females with PTSD. We discovered a PACAPergic projection from the basomedial amygdala (BMA) to the medial intercalated cells (mICCs) in adult mice.

## Introduction

Given the very high prevalence of stress-related pathology in society, it is essential to understand the cellular and circuit mechanisms underlying emotion dysregulation (School, 2007). One theory of heightened fear in post-traumatic stress disorder (PTSD) is because of inappropriate inhibitory control over fear, leading even mild reminders of trauma to trigger strong symptoms and reduced propensity to extinguish acquired fear (Fanselow et al., 1988; Rosen and Schulkin, 1998; Grillon and Morgan, 1999; Milad et al., 2006; Jovanovic et al., 2010).

The amygdala and its associated structures play a key role in processing and reacting to emotional stimuli, and it is known to be involved in PTSD (Stevens et al., 2017). The cortex-like regions of the amygdala proper (lateral and basal nuclei of the basolateral amygdala complex [BLA]) receive sensory information from neocortex and thalamus (Swanson and Petrovich, 1998). Plasticity within these nuclei supports associative learning about sensory information pertinent to positive and negative affect and supports processes, such as Pavlovian fear conditioning (Falls et al., 1992; Fanselow and Kim, 1994; Blair et al., 2001). On the other hand, generation of most fear-related behaviors is initiated by the nearby medial portion of the striatal-like central nucleus (CN) (Swanson and Petrovich, 1998). There are several routes of communication between the amygdala and CN, only some of which are direct (Pare et al., 2004). Indirect microcircuits include relays in the lateral portion of the CN (Haubensak et al., 2010; Li et al., 2013) as well as clusters of GABAergic cells that lie in the capsule separating BLA and CN (Pare et al., 2004). There is currently only limited information about which specific aspects of fear learning are selectively served by these separable microcircuits. The capsular, or medial intercalated cell clusters (mICCs), appear to play a role in fear extinction, the loss of fear responses to a stimulus that previously triggered fear because of repeated exposure without any aversive consequences. A majority of the mICCs express μ-opioid receptors on their cell bodies, and selective ablation of these neurons partially eliminates recall of fear extinction (Likhtik et al., 2008). Whether or not the mICCs are involved in other aspects of fear, such as acquisition, recall, and generalization, is unknown. Therefore, the present experiments combined a contextual fear conditioning task that allowed us to interrogate each of these behaviors with a novel approach to target the medial ICCs dissecting specific neuronal pathways expressed in this circuitry.

The neurotransmitters and neuromodulators that the basolateral complex utilizes to communicate with the mICCs are partially unknown (Mańko et al., 2011; Asede et al., 2015). Pituitary adenylyl cyclase-activating peptide (PACAP) and its G-protein coupled receptor, PAC1, are expressed in brain areas involved in emotion and arousal, including the amygdala and mICCs. Polymorphisms in either the PACAP or PAC1 receptor locus have been linked to PTSD symptom severity, with this genetic link especially strong in females with PTSD (Ressler et al., 2011). PACAP enhances contextual fear consolidation and extinction, and enhances excitatory synaptic transmission in BLA to lateral CN circuit (Cho et al., 2012; Schmidt et al., 2015; Meloni et al., 2016; Kirry et al., 2018). We investigated PACAP/PAC1 functions within the amygdala microcircuitry regulating aspects of contextual fear in male and female mice. Using mice for genetically targeting PACAP or PAC1-expressing cells, we identified a microcircuit consisting of PACAP-expressing neurons in the basal medial nucleus of the amygdala (BMA) that project to PAC1-expressing mICCs. BMA is a crucial part of the BLA complex regulating fear and anxiety, but its functions in relation to other amygdala nuclei are not as clearly defined as the BLA (Amano et al., 2011; Adhikari et al., 2015). We discovered that a BMA-mICC microcircuit containing PACAP and PAC1 regulates fear acquisition, generalization, recall, and extinction in a distinct and sex-dependent manner, respectively.

## Materials and Methods

### 

#### Experimental models and subject details

All experimental procedures were conducted in accordance with the guidelines set by the National Institutes of Health and the Institutional Animal Care and Use Committee at the University of California, Los Angeles. All mice were kept on *ad libitum* access to food and water in a light- and temperature-controlled vivarium. Mice (3-4 months) were housed in clear plastic cages (3-5 mice/cage as littermates) in a vivarium with lights on at 7:00 A.M. and off at 7:00 P.M. Experiments were performed between 9:00 A.M. and 3:00 P.M.

##### Mouse lines

Three different mouse lines (male and female sex) used in all aspects of this study. The first was a Tg (ADCYAP1-EGFP) FB22Gsat/Mmucd (RRID:IMSR_MMRRC:012011) reporter mouse line that faithfully expresses EGFP in PACAP-positive neurons. These mice were generated using a bacterial artificial chromosome (RP24-358O1) by the Gene Expression Nervous System Atlas project and obtained from the Mutant Mouse Resource and Research Center. These mice were backcrossed from FVB/NTac to C57BL/6 for at least five generations (Condro et al., 2016). The second mouse line was an ADCYAP1R1^loxP/loxP^ mouse. These mice were generated in a C57BL/6 background with a conditional KO allele (PAC1^loxP/loxP^ mice) through the National Institutes of Health-funded KO mouse project. The third mouse line was an ADCYAP1-2A-Cre mouse line. These mice (ADCYAP1-2A-Cre) target Cre to most populations of PACAP neurons of the brain, including the amygdala [10] (Allen Brain Atlas).

##### Measure of freezing

Freezing is a complete lack of movement, except for respiration (Fanselow, 1980). Freezing was measured using VideoFreeze (Med Associates) that performed real-time video recordings at 18 frames per second. With this program, adjacent frames are compared to provide the grayscale change for each pixel, and the sum of pixels changing from one frame to the next constitutes a momentary activity score. To account for video noise and to approximate scoring by a trained human observer, a threshold is set at 18 activity units so that an instance of freezing is counted when that activity score remains below this threshold for 1 s (Anagnostaras et al., 2001). Percentage freezing = Freezing time/Total time × 100 for a period of interest. Data are presented as mean ± SEM percentages.

Because Med Associates software uses the number of pixels changed across the entire video frame to calculate the amount of freezing, we were not able to use it for automated analysis of freezing for our optogenetic studies. This is because, even when the mice were freezing, the movement in the optogenetic cable was calculated by the software as movement. Hence, for optogenetic behavioral experiments, we analyzed freezing using ezTrack software (Pennington et al., 2019), which enables removal of this cable artifact. In brief, videos were cropped to reduce the influence of optogenetic cables in the upper portion of the FOV. Subsequently, the number of pixels whose grayscale value changed from one frame to the next was calculated. Freezing was then scored when this number dropped below an experimenter-defined threshold for at least 30 frames (1 s). The freezing threshold was determined by visual inspection of the video and by comparing a subset of the results obtained to the results of manual scoring. All cropping and thresholding parameters were identical across sessions. Data are presented as mean ± SEM percentages.

#### Determining BMA to mICCs PACAPergic innervation

##### Immunofluorescence for visualizing expression of PACAP-EGFP neurons and Vesicular glutamate transporter 2 (VGLUT2)

For immunofluorescence labeling, 40 μm coronal brain sections were cut from (ADCYAP1-EGFP) mice (*N* = 4; M = 2, F = 2). Sections were blocked and permeabilized in a solution containing PBS + 10% normal goat serum + 1% BSA + 0.5% Triton X-100 for 1 h. An anti-GFP primary antibody (A11122, Invitrogen), anti-VGLUT2 antibody (ab178538, Abcam), or anti-NeuN antibody (MAB377, Millipore) were diluted 1:500 in PBS + 5% normal goat serum + 1% BSA, and sections were incubated overnight at 4°C, then washed in PBS, and subsequently incubated with an anti-rabbit AlexaFluor-488 (Invitrogen, catalog #A11008, RRID:AB_10563748) or anti-mouse Cy3 (Abcam, catalog #Ab97035, RRID:AB_10680176) secondary antibodies diluted 1:400 for 2-4 h at room temperature. Sections were washed in PBS and mounted on slides, and coverslipped with Prolong Gold Antifade Reagent (Invitrogen). Fluorescence images were acquired with a Keyence widefield microscope (BZ-X710).

##### Intersectional viral method for labeling PACAPergic neurons from BMA to mICCs

Intersectional viral technique uses conditional single-AAV system to express opsins or florescence depending on multiple cell-type features using Boolean logical operations allowing labeling cells that are genetically wired (Fenno et al., 2014). Therefore, to further validate the existence of PACAPergic projections from BMA to mICCs, we used an intersectional viral labeling method. For this, we used a hEF1α-LS1L-mCherry-IRES-flpo virus (Harvard Vector Core), with either AAV5-EF1a-fDIO-ChR2-eYFP-WPRE or AAV5-EF1a-fDIO-eYFP-WPRE (UNC Vector Core) viruses. ADCYAP1-2A-Cre mice (*N* = 3) went through stereotaxic surgeries with BMA coordinates: LM: ± 3.25; AP: −1.7; DV: −4.75) and mICCs coordinates: LM: ± 2.7; AP: −1.06; DV: −4.2. We first microinfused hEF1α-LS1L-mCherry-IRES-flpo into the mICCs and the AAV5-EF1a-DIO-hChR2(H134R)-mCherry virus into the BMA. The hEF1α-LS1L-mCherry-IRES-flpo virus is a (Cre) recombinase-dependent and retrogradely transporting virus, so when injected into the mICCs of ADCYAP1-2A-Cre mice, it only expresses in PACAP-containing neurons as they express Cre in the mICC terminal where it is injected. This virus in turn contains flippase (FLP) recombinase in its sequence. So, we then microinjected a FLP-dependent AAV5-EF1a-fDIO-ChR2-eYFP-WPRE or AAV5-EF1a-fDIO-eYFP-WPRE virus into the BMA. This allows the ChR2 or eYFP (control) to be expressed specifically in PACAPergic neurons that project from the BMA to mICCs. The volume of viral injections in mICCs was 0.1 μl over 2 min, and in the BMA was 0.3 μl over 6 min with 10 min of diffusion time for each infusion. Following the surgical procedure, in all experiments, mice were allowed to recover for 21 d to allow viral transduction.

##### Visualizing expression of PACAPergic neurons

After recovery from surgery, mice were killed, brains extracted, and 40 μm brain sections were cut. Sections were mounted on slides and coverslipped with Prolong Gold Antifade Reagent (Invitrogen). Fluorescence images were acquired with a Keyence imager. We also conducted immunohistochemistry on some slices using an antibody against FoxP2 protein, which is highly expressed in the mICCs and used as a marker of labeling in this region.

#### *In vivo* optogenetic stimulation of BMA-mICC PACAPergic neurons and fear behaviors

##### Intersectional viral method for expressing ChR2 specifically in PACAPergic neurons

We wanted to determine whether altering the activity of PACAPergic neurons that innervate the mICCs changes fear behavior. A previous study showed that the *ex vivo* optogenetic method can be used to analyze and study the role of intercalated cells in regulating fear behaviors, so we used optogenetics to answer our question (Bosch et al., 2016). For these experiments, we used the ADCYAP1-2A-Cre mice and conducted the same intersectional virus labeling strategy described previously. Briefly, we injected the Cre-dependent hEF1α-LS1L-mCherry-IRES-flpo in the mICCs and AAV5-EF1a-fDIO-ChR2-eYFP-WPRE or AAV5-EF1a-fDIO-eYFP-WPRE in the BMA for specifically expressing ChR2 in PACAPergic neurons that project from BMA to ICCs. Two 200-µm-diameter optic fibers were also implanted bilaterally above mICCs or BMA (available from Prizmatix) and cut at the length of 4.6 or 5 mm, respectively. The core diameter of the fibers was 250 µm and an outer diameter of 275 µm. The numerical aperture (NA) was 0.66. For the stimulation, the optical fiber was connected to an LED-emitting blue light (473 nm, 20 Hz train of 25 ms on/off pulses, 10 min duration). Optogenetic stimulation was conducted bilaterally in each mouse throughout the duration of the experiment.

##### Behavioral procedure

The conditioning apparatus is as follows: Mice were run individually in sound- and light-attenuated conditioning boxes (Med Associates) (see Fig. 5). The boxes were equipped with Near Infra-Red Video Fear Conditioning System and could be configured to represent different contexts by changing the internal structure, floor texture, illumination, and odor. Context A (28 × 21 × 21 cm) had a clear Plexiglas back wall, ceiling, and front door with aluminum sidewalls. It also had a grid floor with evenly spaced stainless-steel rods cleaned and scented with 50% Windex. The floor in Context A was connected to a scrambled foot shock generator. Context B had a clear Plexiglas back wall, ceiling, and door with aluminum sidewalls. The chamber was altered by adding a white curved sidewall that extended across the back wall. The floor of Context B consisted of an acrylic white board floor, cleaned and scented with 1% acetic acid solution. The room was illuminated with red light, and the visible overhead lights were turned off in the box.

##### Behavioral design

We designed our behavioral tests to capture effects on all aspects of contextual fear regulation, including acquisition, generalization, recall, and extinction. This design was chosen mainly because mICCs are known to modulate fear extinction, but PAC1 and PACAP modulation could have effects on other aspects of fear. As the circuit mechanisms we are studying have not been examined previously, instead of simply designing a behavioral assay to measure acquisition and extinction, we also added the components to measure generalization and retention in the same animals. For acquisition, we used 5 d of training based on prior work in the laboratory that has shown that 5 d of training is sufficient to produce an asymptotic level of freezing (e.g., Young and Fanselow, 1992).

For each behavior day, mice were lightly restrained, and the optic cable was connected to the indwelling fiber optics in their head and placed in the testing chamber. We tested acquisition, generalization, and extinction of fear as measured by freezing. For optogenetic stimulation studies, we chose to activate throughout sessions to keep the gain-of-function and loss-of-function experiments with PAC1 deletion consistent with each other.

##### Acquisition

For acquisition, we placed the animals in Context A for 4 min 30 s every day around the same time for 5 d. At the fourth minute each day, the mice received a 1 s 0.65 mA shock. After 29 s, they were removed from the chamber and put back in their home cages, where they were housed with littermates. Mice were transported to the laboratory together in their home cages. There was one rest day between acquisition and generalization testing.

##### Generalization Test

The animals were placed in a completely different Context (B) for 4 min 30 s. No shocks were delivered.

##### Recall Test

One day after generalization testing the animals went through fear recall tests in Context A. Mice were placed in the context for 4 min 30 s without any shocks.

##### Extinction Test

For the next 5 d, animals went through fear extinction, again in Context A. Extinction sessions were 30 min long. We measured freezing for the first 4 min, but the animals remained in the chamber for the entire 30 min.

#### Deletion of PAC1 receptors from the mICCs and measurement of fear-related behavior

**Figure 1.**
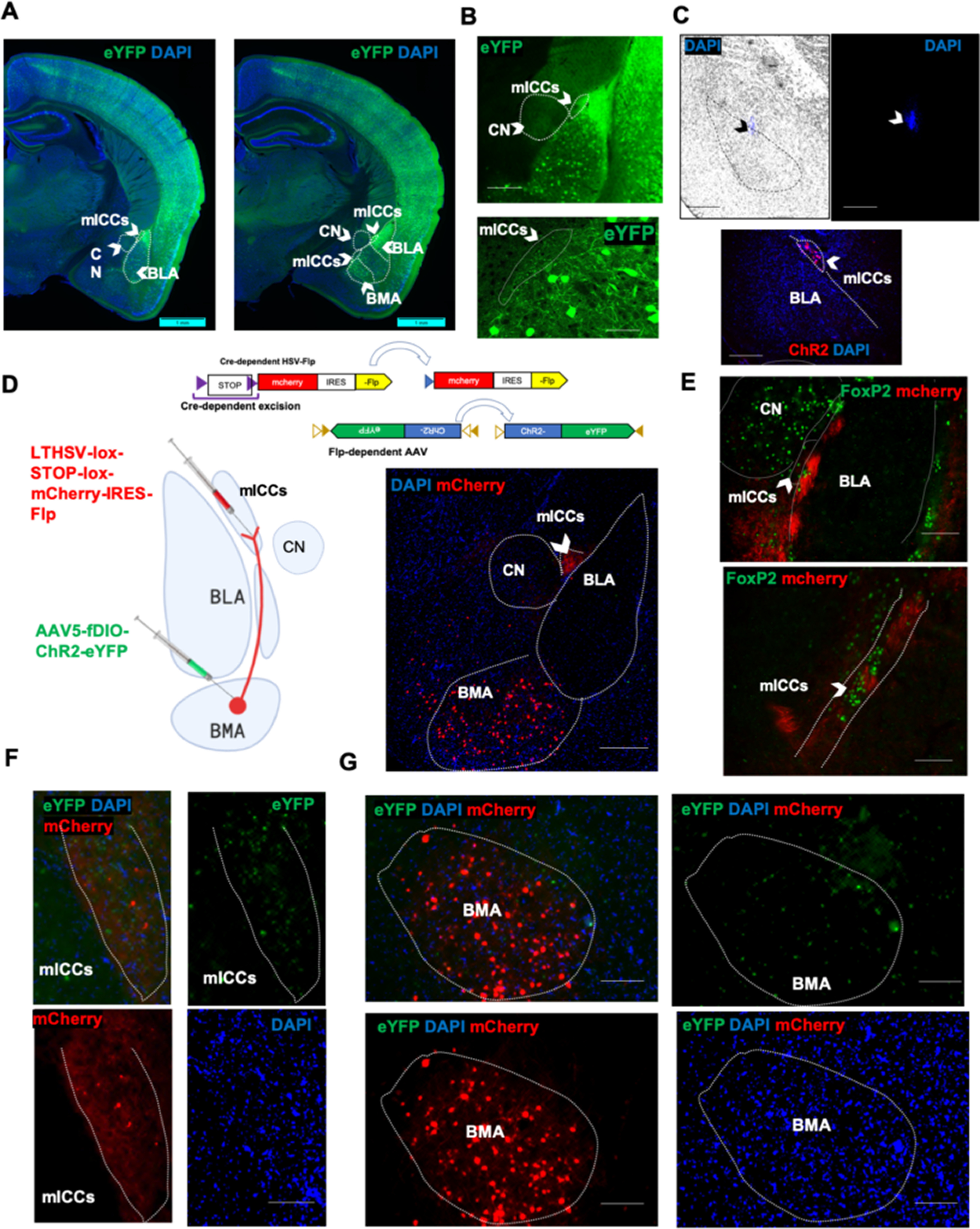
PACAP-expressing neurons in the BMA innervate medial intercalated cells. ***A***, Representative photomicrographs of high-resolution imaging show immunohistochemistry for GFP in ADCYAP1-EGFP mice. PACAPergic neurons are highly expressed in the lateral amygdala and BMA. PACAPergic terminals are observed in the mICCs primarily but not in the central amygdala (CN) (left, right). Green represents PACAP-EGFP. Scale bar, 500 µm. ***B***, Example of section containing the basolateral (BLA) complex and PACAPergic innervation in the mICCs as shown by arrow labeled with PACAP-EGFP (green). Scale bars: top, 100 µm; bottom, 50 µm. ***C***, Top panels, Representative photomicrographs show DAPI (blue) injection into the mICCs using specially constructed glass pipettes as indicated by arrows. Scale bar, 200 µm. Bottom, Representative image shows injection of AAV5-ChR2-mCherry virus into the mICCs. Scale bar, 100 µm. ***D***, Left, Diagram represents the injection strategy for the intersectional approach in ADCYAP1-2A-Cre mice. Top, Representative diagram of the viral constructs that were injected for the intersectional viral injections in ADCYAP1-2A-Cre mice. Top, The Cre-dependent LTHSV-lox-STOP-lox-mCherry-IRES-Flp virus that was injected in the mICCs. Bottom, The AAV5-fDIO-ChR2-eYFP was injected into the BMA. Right, Photomicrograph shows the intersectional virus strategy labeled PACAPergic neuronal in the BMA and terminals in the mICCs (red). ***E***, Representative photomicrographs confirming that the mICC terminals labeled with intersectional virus were in the mICCs by colabeling with the marker FoxP2. FoxP2 was only used here to confirm terminal labeling in the mICCs with the intersectional virus. Arrow indicates the FoxP2 expression in mICCs. ***F***, ***G***, Photomicrographs represent the intersectional viral strategy of injecting LTHSV-lox-STOP-lox-mCherry-IRES-Flp virus into the dorsal mICCs and AAV5-fDIO-ChR2-eYFP into the BMA led to EYFP (green) and mCherry (red) expression from the LTHSV-lox-STOP-lox-mCherry-IRES-Flp and AAV5-fDIO-ChR2-eYFP viruses in the mICCs and BMA, respectively (***F***: mICCs; ***G***: BMA).

For experiments involving deletion of PAC1 receptors, we microinfused AAV2-hsyn-GFP-Cre or AAV2-hsyn-GFP into mICCs using the stereotaxic coordinates LM: ±2.7; AP: −1.06; DV: −4.2. Although it is challenging to precisely target small structures, such as the medial ICCs, we have shown feasibility of confining virus infusion to such a small structure by using a specialized digital stereotax (model 1900, David Kopf Instruments) and pulled glass pipettes that are commonly used for electrophysiological recordings with single-cell resolution. We verified through various methods that we were able to precisely target the mICCs, including DAPI infusion (see Fig. 1*C*), infusion of AAV-expressing mCherry (see Fig. 1*C*), infusion of AAV2-hsyn-GFP (data not shown), and then colabeling our intersectional viral technique with an antibody against FoxP2, protein that is highly expressed in the mICCs (see Fig. 1*E*). Using these methods, we found that viral infusions were constrained by the surrounding capsule if the placement was accurate. The behavioral procedure and design were identical to the optogenetic experiments. The only difference was for measuring freezing, automated Med Associates Videofreeze software was used as described previously.

##### Validation of PAC1 receptor deletion using dual ISH with RNAscope

After the behavioral trials were complete, the mice were killed, and their brains extracted and immediately stored in at −80°C. The brains were sliced at 15 μm in a cryostat and slices containing the amygdala were collected on microscope slides. The deletion of PAC1 receptors was verified using RNAscope for analyzing expression of RNA tissue sections (ACD Biotechne). Briefly, we performed ISH steps following RNAscope 2.5 HD Duplex Assay protocol for fresh frozen sections. After completion of the labeling, sections were coverslipped using Prolong Gold (Thermo Fisher Scientific) with DAPI, and the edges were sealed with clear nail polish. PAC1 mRNA puncta counts were conducted in 4 serial sections containing the mICCs that started at the same rostral plane that were captured with a 40× objective. mICCs frame was 25 μm × 25 μm size. Analysis of dots was conducted using FIJI software. For each image, DAPI+PAC1 mRNA and DAPI+GFP were analyzed separately. For counts, DAPI-positive cells and PAC1 RNA dots were counted separately and expressed as mean grain count.

#### Measurement of CFOS expression after *in Vivo* optogenetic stimulation of BMA-PACAP neurons that innervate the mICCs

##### Viral surgeries and *in vivo* optogenetic stimulation

For these experiments, we used the same intersectional approach described above for labeling PACAPergic neurons projecting from BMA to mICCs by infusing hEF1α-LS1L-mCherry-IRES-flpo into the mICCs (*N* = 4, 2 males and 2 females) and AAV5-EF1a-fDIO-ChR2-eYFP-WPRE or AAV5-EF1a-fDIO-eYFP-WPRE into the BMA. A 200-µm-diameter optic fiber was also implanted bilaterally above BMA (available from Prizmatix) and cut at the length of 5 mm. The core diameter of the fibers was 250 μm, and the outer diameter was 275 µm. The NA was 0.66. After 21 d, mice were anesthetized with isoflurane, and the optical fiber was connected to an LED-emitting blue light (473 nm, 20 Hz train of 10 ms on/off pulses, 10 min duration). Optogenetic stimulation was conducted unilaterally, and the hemisphere of stimulation was counterbalanced between mice. Ninety minutes following the simulation, mice were killed, and brains extracted, cryoprotected, and frozen. During the 90 min, the mice were in their home cages without anesthesia.

##### Immunohistochemistry for measuring CFOS expression

The brains were processed for CFOS immunohistochemistry. First, 40 μm coronal sections containing the amygdala were collected serially. On day 1, tissue sections were washed in 1× TBS 3 times for 5 min, then blocked in 1 ml of 1× TBS with 5% normal donkey serum, 0.1% BSA, and 0.3% Triton-X for 1 h. Then the tissue sections were incubated overnight at 4°C with the primary goat polyclonal to cfos (1:500, 24 h, Abcam; RRID:SCR_012931) primary antibody. According to the manufacturer, this antibody is a “synthetic peptide conjugated to Blue Carrier Protein by a Cysteine residue linker corresponding to the internal sequence amino acids 283-295 of Human c-Fos (NP_005243.1).” On the second day, the sections were washed in 1× TBS 3 times 5 min each and then incubated in the Alexa-488 donkey anti-goat secondary antibody (1:200, Invitrogen) for 2 h at room temperature. After washing with 1× TBS for 3 times for 5 min each, tissue sections were mounted on glass slides and coverslipped using Prolong Gold (Thermo Fisher Scientific) with DAPI, and the edges were sealed with clear nail polish. Positive cfos immunolabeling was analyzed and quantified in brain sections containing the mICCs.

##### Cell counts for cfos experiments

Sections in the rostrocaudal extents of the BMA (bregma −1.06 to −2.3) were collected and processed for immunohistochemistry or RNAscope+immunohistochemistry, as described. The numbers of cfos^+^ and VGLUT2^+^ cells were manually counted in the region of the mICCs by two trained researchers that were blind to the treatment conditions. For VGLUT2, all PACAP cells, VGLUT2 cells, and PACAP+VGLUT2 cells were manually counted first for the tissue sections from PACAP-EGFP mice with VGLUT2 and GFP immunohistochemistry. For tissue sections from WT mice, with immunohistochemistry for VGLUT2 and RNAscope for PACAP mRNA, VGLUT2-positive cells, and colocalization with PACAP were counted manually using Fiji (National Institutes of Health) software. For RNAscope ISH and immunohistochemistry experiments, we counted PACAP-labeled cells in BMA and assessed colocalization with either VGLUT2 using Fiji software. For RNAscope, at least 5 PACAP mRNA puncta were counted that were in close proximity to a DAPI-labeled nucleus. For both, immunohistochemistry and RNAscope experiments, three or four sections from each animal were counted.

#### Electrophysiological recordings of mICC neuronal activity and *ex vivo* optogenetic stimulation of PACAPergic neurons that innervate mICCs

##### Electrophysiology and *ex vivo* optogenetics

For *ex vivo* electrophysiology experiments, we infused in the BMA of the ADCYAP1-2A-Cre mice a Cre-dependent AAV5-EF1a-DIO-hChR2(H134R)-mCherry virus (UNC Vector Core) (*N* = 6; M = 3, F = 3). After a 21 d recovery from surgery, animals were deeply anesthetized with isoflurane, and decapitated amygdala slices were prepared. The brains were placed in ice-cold modified aCSF, containing the following (in mm): 194 sucrose, 30 NaCl, 4.5 KCl, 1 MgCl_2_, 26 NaHCO_3_,1.2 NaH_2_PO_4_, and 10 D-glucose, and cut into 300-μm-thick coronal slices containing the ICC layer of the amygdala. The slices were then allowed to equilibrate for 30 min at 32°C-34°C in normal aCSF containing the following (in mm): 124 NaCl, 4.5 KCl, 2 CaCl_2_, 1 MgCl_2_, 26 NaHCO_3_, 1.2 NaH_2_PO_4_, and 10 D-glucose, continuously bubbled with a mixture of 95% O_2_/5% CO_2_, stored at room temperature in the same buffer, and used for experiments within 6 h of slice preparation.

Electrophysiological methods were described previously (Tong et al., 2014; Octeau et al., 2018). Cells were visualized with infrared optics on an upright microscope (BX61WI, Olympus). pCLAMP10 software and a MultiClamp 700B amplifier were used for electrophysiology (Molecular Devices). For these recordings, the intracellular solution in the patch pipette contained the following (in mm): 135 potassium gluconate, 3 KCl, 0.1 CaCl_2_,10 HEPES, 1 EGTA, 8 Na_2_-phosphocreatine, 4 Mg-ATP, 0.3 Na_2_-GTP, pH 7.3, adjusted with KOH and filtered with a 0.2 µm syringe filter. For biocytin labeling, 2 mg/ml biocytin (Tocris Bioscience) was dissolved in the intracellular solution, and cells were dialyzed for 20 min. For all recordings, the patch-pipette tip resistance was ∼5 mΩ. The initial access resistance was <25 mΩ for all cells; and if this increased by >5 mΩ, the cell was discarded.

We identified mICCs by their somatic morphology and location in the dorsal ICC nucleus, elevated membrane resistance, and the presence of a slowly accommodating inward current on hyperpolarization, in agreement with past work (Asede et al., 2015). The concentration of drugs applied onto brain slices via bath perfusion was as follows: 10 μm CNQX (Cayman Chemical), 250 nm PACAP 6-38 (Tocris Bioscience), 20 μm bicuculline (Cayman Chemical), and 0.5 μm TTX (Cayman Chemical). All compounds were stored at −20°C as stock solutions and diluted in aCSF just before use. ChR2-mediated responses were evoked by 470 nm light flashes from an LED source (Sutter Instrument) at power of 0.025 mW/mm^2^ and for a flash duration of 25 ms each.

##### Viruses

For the intersectional method, we used hEF1α-LS1L-mCherry-IRES-flpo (MIT and Harvard Vector Core) and AAV5-EF1a-fDIO-ChR2-eYFP-WPRE or AAV5-EF1a-fDIO-eYFP-WPRE (UNC Vector Core) constructed by Rachel Neve and Karl Deisseroth, respectively. For *ex vivo* electrophysiology experiments, we used Cre-dependent AAV5-EF1a-DIO- hChR2(H134R)-mCherry virus (UNC Vector Core). For deletion of PAC1 receptors, we used the AAV2-hsyn-GFP-Cre or AAV2-hsyn-GFP (UNC Vector Core).

#### Microscopy for all experiments

The tissue sections were analyzed using a Keyence BZ-X700-All-in-One Fluorescence Microscope. Images were analyzed with Fiji image processing software (National Institutes of Health; RRID:SCR_002285). Images were converted to binary mode (black-and-white image). For *ex vivo* studies, a confocal microscope was used for analyzing the expression of biocytin-filled cells in the mICCs.

#### Experimental design and the statistical analyses

To be consistent, for every phase of testing (days 1-14), we measured freezing for the first 4 min of the session. This corresponds to the preshock period on the acquisition days, providing a measure of contextual fear that is not confounded by the unconditional behavioral effects of the shock. For the behavioral experiments in which acquisition and extinction were measured, a three-way ANOVA was used to measure differences in means with two between (sex and group) and one within (day) factors. For the other behavioral experiments, a two-way ANOVA was conducted to measure differences in the means with group and sex as between group factors. Significant effects indicated by the ANOVA were further analyzed with a *post hoc* Bonferroni analysis. The level of significance used for all analyses was *p* < 0.05. For behavior experiments, analysis was conducted using the SPSS statistics software.

For electrophysiology, statistical tests were run in GraphPad Instat 3. Summary data are presented as mean ± SEM. In some of the graphs, the bars indicating the SEM are smaller than the symbols used to represent the mean. In all cases, the individual *n* numbers are reported on a scatter plot and defined for each experiment. For each set of data to be compared, we determined within GraphPad Instat whether the data were normally distributed or not. If they were normally distributed, we used parametric tests. If the data were not normally distributed, we used nonparametric tests. Paired and unpaired Student's *t* tests (as appropriate) and two-tailed Mann–Whitney or Wilcoxon tests were used for most statistical analyses with significance declared at *p* < 0.05, but stated in each case with a precise *p* value. When the *p* value was <0.0001, it is stated as such to save space on the figure panels and text. *N* is defined as the numbers of cells or mice throughout on a case-by-case basis depending on the particular experiment; the unit of analysis is stated in each figure or figure legend. Where appropriate, key statistics are also reported in the text. No data points were excluded from any experiment.

## Results

### PACAP-expressing neurons in the BMA innervate the medial ICCs

We examined EGFP expression in the ADCYAP1-EGFP mice, which restricts EGFP expression to PACAP-expressing neurons (Condro et al., 2016). Although distributed broadly in the amygdala, EGFP^+^ cells were enriched in the lateral and basomedial nucleus (BMA) subregion with fibers in the mICCs (Fig. 1*A*,*B*). We evaluated the local PACAPergic efferents in the mICCs and found a high innervation of dorsal and ventral mICCs by PACAPergic neurons (Fig. 1*A*,*B*). By comparison, we found little to no innervation of the CN by EGFP^+^ neurons (Fig. 1*A*,*B*). The pattern of expression corresponds with PACAP mRNA expression, which is high in the BMA and some expression in the mICCS (Allen Brain Atlas Mouse Brain ISH). To specifically target the mICCs, we first injected DAPI using glass pipettes. While mICCs are difficult to target because of their small size, we were able to localize our DAPI injections to these cells into the capsule of mICCs (Fig. 1*C*). We also confirmed that our injections would be restricted to the mICCs by injecting AAV5-EF1a-DIO-hChR2(H134R)-mCherry into the mICCs and were able to restrict the injection in the mICCs by this method (Fig. 1*C*). We then wanted to determine whether monosynaptic PACAPergic projections in the mICCs arise from the BMA. Using intersectional approach as described by Fenno et al. (2014), we injected a retrogradely trafficked Cre-dependent HSV virus (hEF1α-LS1L-mCherry-IRES-flpo) into the mICCs (dorsal portion) and a Flp-dependent AAV5-EF1a-fDIO-ChR2-eYFP-WPRE into the BMA of ADCYAP1-2A-Cre mice. Adcyap1-2A-Cre mice express Cre specifically in PACAP-containing neurons. With the intersectional approach, the HSV virus, which is Cre-dependent and expresses FLP, retrogradely transports allowing expression specifically in PACAPergic neurons in the BMA. The AAV virus in turn is FLP-dependent, so therefore expresses only in neurons that have FLP. This allows labeling of specific projections from the BMA to mICCs (Fig. 1*D*). Our analysis showed that mCherry expressing soma were present in the BMA and fibers in the mICCs (Fig. 1*D–G*). To confirm, mCherry fiber localization in the mICCs, we also colabeled the sections with antibody against FoxP2, a marker for mICCs and found that the viral labeling overlapped with FoxP2 (Fig. 1*E*). Thus, using two different approaches (in Adcyap1-EGFP and Adcyap1-2A-Cre mouse lines), we confirmed that mICCs receive PACAPergic innervations and some of those innervations arise in the BMA.

### *In vivo* optogenetic activation of PACAP-expressing neurons in the BMA enhances expression of cfos in the mICCs

**Figure 2.**
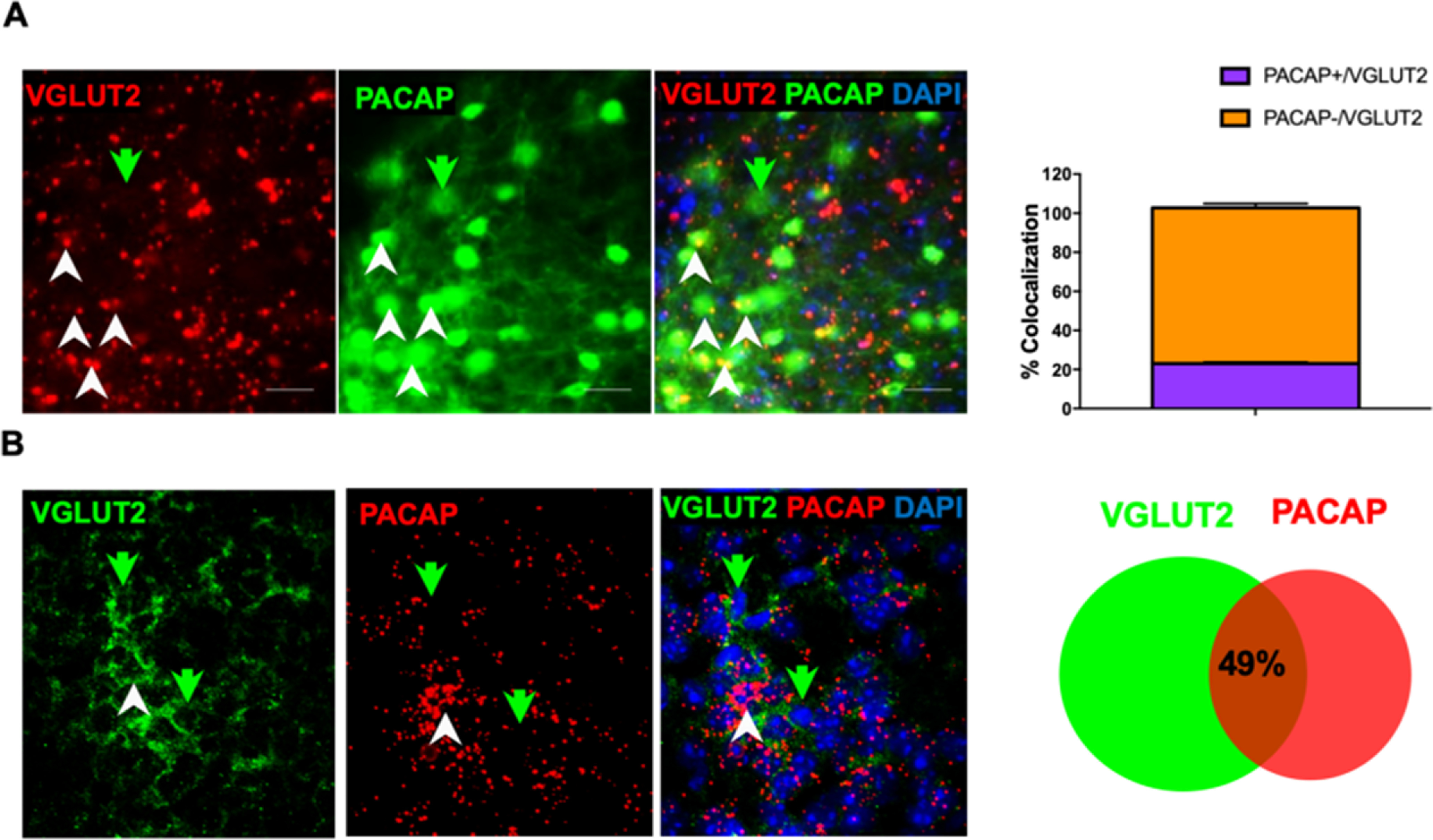
Immunohistochemistry and ISH analysis and quantification of overlapping PACAP- and VGLUT2-expressing cells in the BMA. ***A***, Representative photomicrographs from tissue sections containing the BMA from PACAP-EGFP mice represent expression of VGLUT2 (red) and PACAP-EGFP (green) processed with immunohistochemistry for GFP and VGLUT2. White arrows indicate overlapping VGLUT2 and PACAP signals. Green arrow indicates an example of PACAP cell without VGLUT2 expression. Scale bar, 25 µm. Right, Quantification of overlap (*N* = 6: 3 M and 3 F; df = 5). ***B***, Representative photomicrographs from tissue sections containing the BMA from WT mice represent overlapping VLGUT2 (green) and PACAP mRNA (red) fluorescence in the BMA. Tissues were processed with RNAscope technique for PACAP mRNA and then immunohistochemistry for VGLUT2. Right, Venn diagram represents quantification of overlap between VGLUT2 and PACAP mRNA (*N* = 5: 2 M and 3 F; df = 4).

Next, we wanted to validate whether the mICCs projecting BMA PACAPergic neurons are glutamatergic. We first characterized colocalization of PACAPergic neurons with glutamatergic neurons with immunohistochemistry using an antibody against VGLUT2, a marker for glutamatergic neurons, and GFP in tissue sections containing the BMA from Adcyap1-EGFP mice (*N* = 6, 3 M and 3 F; Fig. 2*A*). We analyzed the percentage of overlap between VGLUT2-expresising neurons and PACAP. For this, we first counted the number of VGLUT2^+^ and PACAP^+^ cells separately in the same area of BMA. We then counted the VGLUT2^+^ that were either PACAP^+^ or PACAP^–^. Then we expressed these counts as fraction of overall VGLUT2 cells and expressed as percentage (paired *t* test PACAP^+^/VGLUT2 = 23 ± 0.006%; PACAP^–^/VGLUT2 = 77 ± 0.015%; *p* = 0.0001, *t*_(5)_ = 26.15). Our analysis showed that ∼23% of VGLUT2 cells in BMA express PACAP-GFP. We further conducted ISH for PACAP using the RNAscope technology and immunohistochemistry using an antibody against VGLUT2 in tissue sections containing the BMA in a separate group of WT mice (*N* = 8: 4 M and 4 F; Fig. 2*B*). We analyzed the percentage of overlap between VGLUT2-immunoreactive cells with PACAP mRNA puncta. We found that PACAP mRNA puncta were present throughout the BMA. For our analysis, we first counted VLGUT2^+^ cells. Then we counted the PACAP mRNA puncta surrounding VLGUT2^+^ cells by setting the criterion of counting >5 puncta surrounding the VGLUT2 and DAPI-expressing cell. This analysis revealed that ∼49 ± 3% VGLUT2 cells expressed PACAP mRNA (Fig. 2*B*). Our analysis shows that there is a significant difference in the expression of PACAP mRNA and PACAP protein expression within the BMA, with higher expression of the PACAP mRNA overlapping with VGLTU2-containing neurons.

### *In vivo* optogenetic activation of PACAP-expressing neurons in the BMA enhances expression of cfos in the mICCs

**Figure 3.**
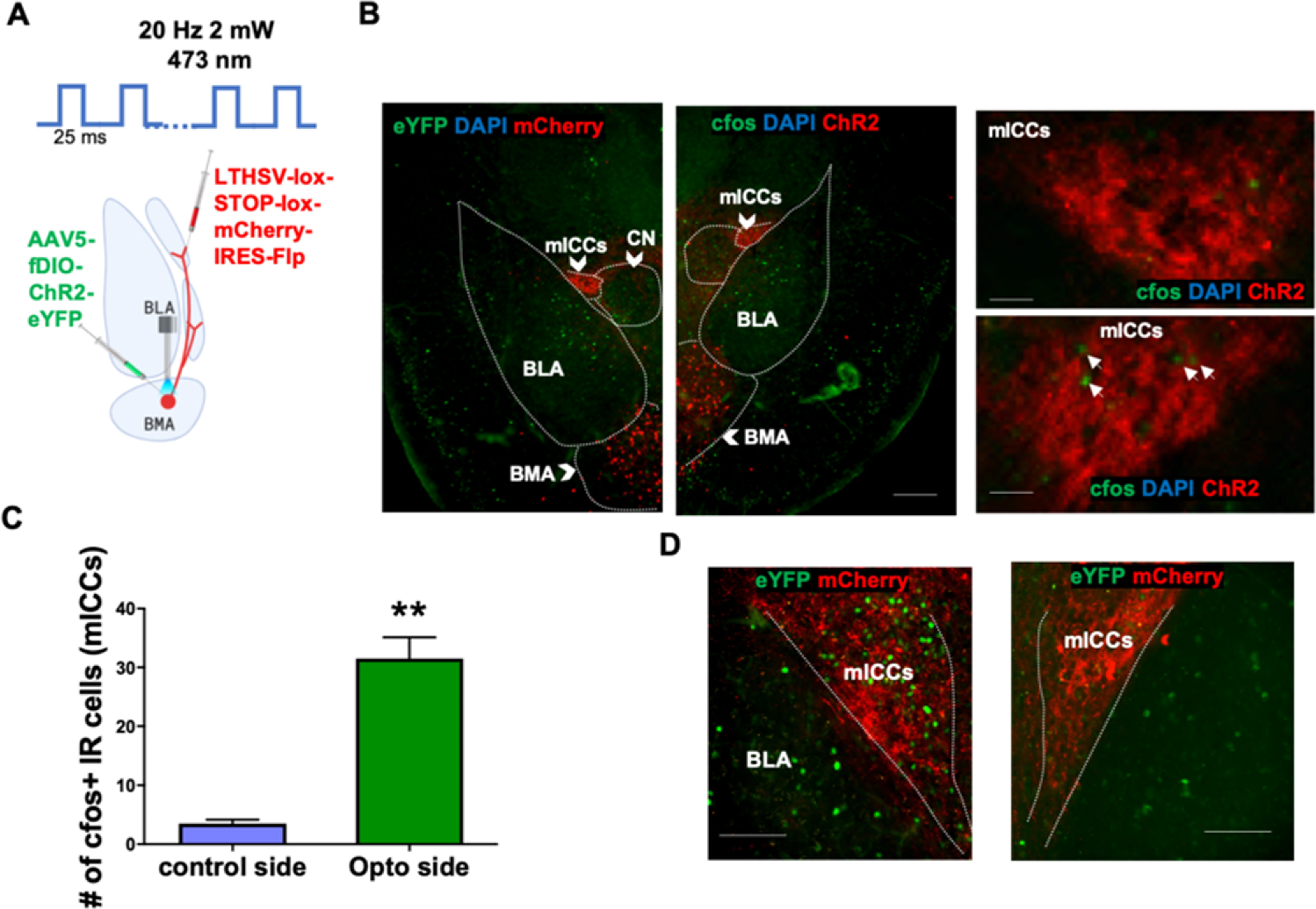
*In vivo* optogenetic activation of PACAP-expressing neurons in the BMA enhances expression of cfos in the mICCs. ***A***, Diagram represents optogenetic stimulation strategy in the BMA in anesthetized Adcyap1-2A-Cre mice. Cre-dependent LTHSV-lox-STOP-lox-mCherry-IRES-Flp virus that was injected in the mICCs and the AAV5-fDIO-ChR2-eYFP was injected into the BMA. Optogenetic stimulation was conducted in unilateral hemispheres, and control stimulation was in the opposite hemisphere of the same mice. Stimulation parameters are shown above the diagram on the top. ***B***, Representative photomicrographs of hemispheres containing mICCs and other regions represent cfos expression after optogenetic stimulation in the BMA of anesthetized mice. Arrows indicate cfos expression. Scale bars, 100 µm. ***C***, Graphical representation represent number of cfos-positive cells in mICCs. Right panels, Representative images represent cfos immunoreactivity expression in the mICCs in the hemisphere that was optogenetically stimulated and the control (*N* = 4: 2 M, 2 F; df = 3, *p* < 0.001). ***D***, Representative photomicrographs of mICCs represent cfos expression in the hemisphere where optogenetic stimulation occurred (left) and the hemisphere where the stimulation was absent (right). Scale bars, 100 µm. *significant *p* value.

Next, we wanted to determine the functional effect of stimulating PACAPergic projections that innervate the mICCs. We chose to measure changes in expression of cfos immunoreactivity after optogenetic stimulation of the BMA-mICC PACAPergic pathway. Using the same intersectional approach as described above in the Adcyap1-2A-Cre mice, we first injected the Cre-dependent HSV virus (hEF1α-LS1L-mCherry-IRES-flpo) into the mICCs (dorsal portion) and a Flp-dependent AAV5-EF1a-fDIO-ChR2-eYFP-WPRE into the BMA of Adcyap1-2A-Cre mice in both hemispheres. We bilaterally implanted optic fibers targeting the BMA (Fig. 3*A*). After recovery and viral expression, we performed *in vivo* optogenetic stimulation of the BMA in anesthetized mice in one hemisphere and analyzed changes in expression of cfos immunoreactivity in the mICCs of that hemisphere comparing it with the opposite side. For this, we conducted immunohistochemistry for labeling cfos in tissue sections containing the BMA and mICCs using an antibody against cfos. For analysis, we counted the absolute number of cfos^+^ cells in the mICCs in both hemispheres. Only distinct soma-like puncta were counted for the analysis. Optogenetic stimulation of ChR2-contaning cells in the BMA significantly enhanced expression of cfos in the mICCs in the hemisphere with optogenetic stimulation compared with mICCs of the hemisphere without optogenetic stimulation (*N* = 4: 2 M and 2 F; paired *t* test; *t*_(3)_ = 7.816, *p* = 0.0024; Fig. 3*A–D*). We used the Paxinos and Watson Brain Atlas to determine the anatomic boundaries between CN and mICCs for analysis of cfos and restricted it to the mICCs in the dorsal region. These results show that altering activity of PACAPergic neurons in the BMA modulates activity of neurons in the mICCs, represent a functional effect on neuronal activity in the mICCs.

### *Ex vivo* optogenetic stimulation of the BMA-ICC PACAPergic pathway enhances EPSCs in the mICCs that is further enhanced by application of a PAC1 receptor antagonist

**Figure 4.**
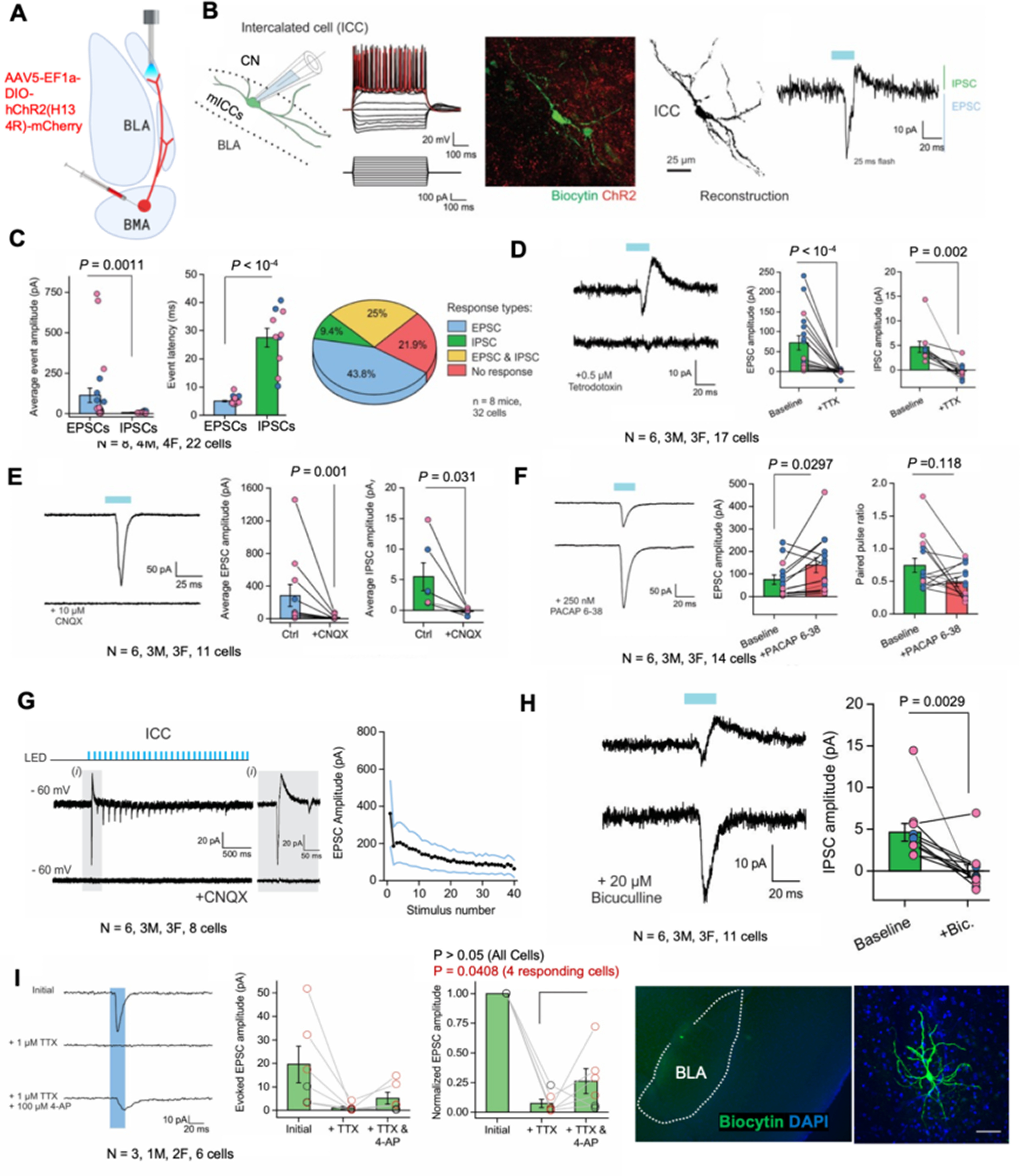
*Ex vivo* optogenetic stimulation of BMA-ICCs PACAPergic pathway enhances EPSCs in the mICCs. ***A***, Diagram represents the location of the ChR2-expressing cells within the BMA that innervate the mICCs. ***B***, Left, Diagram represents the whole-cell patch clamp of a neuron in the mICCs. Whole-cell electrophysiological properties of mICCs with the current injection parameters depicted below. Middle, Example of a biocytin-filled mICC neuron (left) and reconstruction (right). Right, Example trace represents the electrophysiological responses of mICC neurons relative to the LED flash. In this example, the neuronal response comprises of an EPSC followed immediately by an IPSC response. Scale bar, 25 µm. ***C***, Graphs represent the EPSC and IPSC amplitudes (left), response latency of either EPSC or IPSC events (middle), and pie chart represents the classification of response types from mICCs. Majority of mICC neurons showed EPSCs (*N* = 8, Mann–Whitney *U* test, *p* < 0.01). Pink dots represent females. Blue dots represent males. ***D***, Representative current trace (left) from mICC neuron in response to single flashes with or without TTX applied. Average EPSC (middle) or IPSC (right) amplitude in response to single flashes with or without TTX are also shown (*N* = 8, Mann–Whitney *U* test, *p* < 0.002). Pink dots represent females. Blue dots represent males. ***E***, Left, Representative current trace from mICC neuron in response to single flash with or without PACAP 6-38 application. Average EPSC amplitude (middle) or paired-pulse ratio (right) in response to single flashes with or without PACAP 6-38 is also shown (*N* = 6, *p* < 0.05). Pink dots represent females. Blue dots represent males. ***F***, Left, Representative current trace from mICC neuron in response to single LED flash with or without CNQX application. Average EPSC (middle) or IPSC (right) amplitudes in response to single LED flash with or without CNQX (*N* = 6, *p* < 0.05). Pink dots represent females. Blue dots represent males. ***G***, Representative current traces (left) from mICCs in response to trains of blue light flashes with or without CNQX present and average EPSC amplitude (right) at each flash for a train of 40 flashes. Inset, Zoomed in trace with both EPSCs and IPSCs present. ***H***, Representative current trace (left) from mICCs in response to single flashes with or without bicuculline application. Average IPSC amplitude (right) in response to single flashes with or without bicuculline (*N* = 6, *p* < 0.05). Pink dots represent females. Blue dots represent males. ***I***, Representative current traces in mICCs in response to light stimulation of ChR2 in PACAPergic neurons from BMA-mICCs in response to single flashes in the presence of 1 μm TTX and 100 μm TTX + 4-AP. Average evoked EPSC amplitudes (middle) and normalized evoked EPSC amplitudes (right) in response to single flashes with TTX and TTX + 4-AP (*N* = 3, *p* < 0.05). Pink dots represent females. Blue dots represent males. ***J***, Representative image of one example neuron filled with biocytin in the mICCs after electrophysiological recordings. Inset, Confocal image of a biocytin-filled mICC neuron that has morphologic properties and processes that looks like a medium spiny neuron. Scale bar, 30 mm.

Next, we sought to characterize the electrophysiological properties of mICC neurons when PACAPergic projections to this region were optogenetically stimulated. We specifically tested whether stimulation of BMA PACAPergic terminals changes synaptic activity of mICC neurons. For this, we performed *ex vivo* electrophysiological recordings in combination with optogenetic stimulation of BMA PACAPergic fibers expressing ChR2 (Fig. 4*A*). We injected AAV5-EF1a-DIO-hChR2(H134R)-mCherry virus into the BMA of Adcyap1-2A-Cre mice, which expresses Cre in PACAP-containing neurons (both males and females) (Fig. 4*A*,*B*). This allowed expression of ChR2 in the efferent pathways of the BMA containing PACAP. We then performed whole-cell patch-clamp recordings from the dorsal mICC region (Fig. 4*B*). The mICC neurons showed a sagging current on hyperpolarization and action potentials in response to stepwise changes in current (Fig. 4*B*). For all electrophysiological experiments, we confirmed that the recording sites were within the mICCs by filling the recorded neurons with biocytin and confirming the appropriate location of the neurons *post hoc* (Fig. 4*B*,*J*). The electrophysiological and morphologic properties of mICC neurons in our studies matched previously described properties of mICC neurons (Huang et al., 2014; Asede et al., 2015; Bosch et al., 2016).

Optogenetic stimulation of PACAPergic neurons from BMA was conducted with a 473 nm light pulsed with a 20 Hz train of 25 ms single pulse or multiple pulses using an LED (Fig. 4*B*). The stimulation parameter was based on a previous study published in the laboratory (Hersman et al., 2017). A single flash produced EPSCs in a majority of mICC neurons (Fig. 4*C*). In some cases, EPSCs were followed by IPSCs (Fig. 4*C*). Of 32 recorded neurons, 43.8% showed EPSCs, 9.4% showed IPSCs, 25% showed EPSCs followed by IPSCs, and 21.9% of cells showed no response (*N* = 8, 22 cells). Mann–Whitney, unpaired test showed that average amplitude of EPSCs was enhanced compared with IPSCs (*U* = 187; *p* = 0.0011; Fig. 4*C*). For the neurons that showed EPSCs followed by IPSCs, unpaired *t* test showed that the IPSC event latencies were much longer compared with the EPSCs (*t*_(30)_ = 10.215; *p* = 0.0001; Fig. 4*C*). Wilcoxon matched pairs, two-tailed test showed that application of TTX completely abolished EPSCs and IPSCs (EPSCs; W_(17)_ = −153; *p* = 0.0001; IPSCs; W_(10)_ = 55; *p* = 0.002; Fig. 4*D*). Wilcoxon matched pairs, two-tailed test also showed that optogenetic light-evoked EPSCs and IPSCs were blocked by the application of CNQX in both cases, indicating that the BMA neurons release glutamate (*N* = 6; 11 cells [EPSCs]; 6 cells [IPSCs]; EPSCs; W_(11)_ = −66; *p* = 0.001; IPSCs; W_(6)_ = 21; *p*= 0.0313; Fig. 4*E*). Paired *t* test showed application of the PAC1 receptor antagonist peptide, PACAP 6-38, in the bath significantly enhanced EPSCs (*N* = 6; 14 cells; *t*_(13)_ = 2.441; *p* = 0.0297; Fig. 4*F*). However, Wilcoxon matched pairs, two-tailed test showed that application of PACAP 6-38 did not alter paired-pulse ratio (W_(14)_ = 51; *p* = 0.1189; Fig. 4*F*).

With bursts of optogenetic stimulation, EPSC amplitude decreased with an increase in stimulus number (Fig. 4*G*; *N* = 6; 8 cells). Wilcoxon-matched pairs, two-tailed test showed that the IPSCs that were observed in some neurons after EPSCs, were blocked by application of bicuculline (*N* = 6; 11 cells; W_(11)_ = 62; *p* = 0.0029; Fig. 4*H*).

In a small subset of neurons, paired *t* test showed that the application of TTX suppressed EPSC responses and TTX + 4-AP application increased EPSC amplitude (*t*_(3)_ = 2.584; *p* = 0.0408; Fig. 4*I*). These results indicate that PACAP neurons produce a predominantly excitatory glutamatergic influence on mICCs, which in turn triggers local GABAergic inhibition between mICCs. Overall, these results show that the PACAPergic neurons from BMA to mICCs are synaptically coupled. These results also suggest that PACAP could be coreleased with glutamate from BMA neurons mitigating the postsynaptic influence of glutamate. In our studies, we did not find any significant differences in the electrophysiological properties between males and females; therefore, the data for males and females were combined but represented as blue and pink colors in Figure 4.

### *In vivo* optogenetic stimulation of BMA PACAPergic input to the mICCs decreases fear recall and increases fear extinction

**Figure 5.**
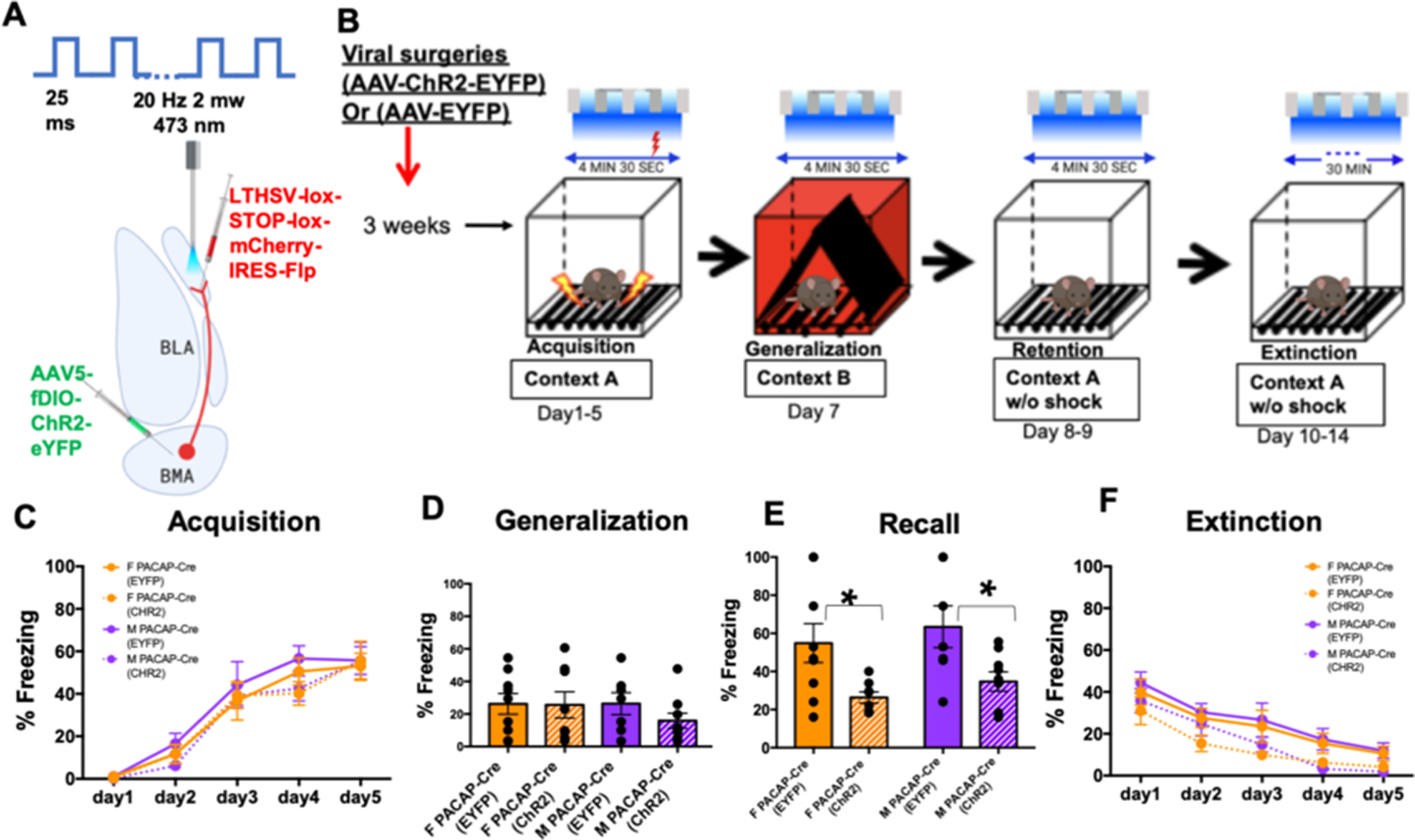
*In vivo* optogenetic stimulation of BMA-mICC PACAPergic inputs decreases fear recall and increases fear extinction. ***A***, Diagram represents the intersectional viral injection strategy in the BMA and mICCs and the optogenetic stimulation strategy and parameters for stimulation. LTHSV-lox-STOP-lox-mCherry-IRES-Flp virus was injected into the dorsal mICCs and AAV5-fDIO-ChR2-eYFP into the BMA. Males (*N* = 8 [ChR2]; *N* = 9 [EYFP]) and females (*N* = 9 [ChR2]; *N* = 7 [EYFP]) were used in these experiments. ***B***, Diagram represents viral injection surgery timeline and behavior protocol. Mice were tested on fear acquisition in Context A, generalization test in Context B, recall test in Context A, and extinction in Context A. Optogenetic stimulation with parameters shown in ***A*** (top) occurred during these test sessions. ***C***, Graphs represent changes in freezing during acquisition in females and males in response to optogenetic stimulation of PACAPergic pathway from BMA-mICCs. There was no significant Sex × Group interaction in fear acquisition: males (*N* = 8 [ChR2]; *N* = 9 [EYFP]) and females (*N* = 9 [ChR2]; *N* = 7 [EYFP]) (*F* = 0.833). ***D***, Graphs represent freezing during fear generalization test in females and males in response to optogenetic stimulation of PACAPergic pathway from BMA-mICCs. The Sex × Group interaction in the generalization test was not significant, indicating that the animals did not differentially generalize fear to a novel context: males (*N* = 8 [ChR2]; *N* = 9 [EYFP]) and females (*N* = 9 [ChR2]; *N* = 7 [EYFP]) (*F* = 0.594). ***E***, Graph represents freezing during fear recall test in females and males in response to optogenetic stimulation of PACAPergic pathway from BMA-mICCs. There was a significant main effect of Group (*N* = 8 [ChR2]; *N* = 9 [EYFP]) and females (*N* = 9 [ChR2]; *N* = 7 [EYFP]) (*F* = 13.08; *p* = 0.001). *Post hoc* comparison indicated that the group that received optogenetic stimulation of the PACAPergic neurons from BMA to mICCs showed a significantly reduced level of freezing compared with the controls. However, there was no significant Group × Sex interaction (*F* = 0.008). ***F***, Graph represents freezing during extinction in females and males after optogenetic stimulation of the PACAPergic pathway from BMA-mICCs. There was a main effect of Group during the extinction test (*N* = 8 [ChR2]; *N* = 9 [EYFP]) and females (*N* = 9 [ChR2]; *N* = 7 [EYFP]) (*F* = 20.128; *p* = 0.0001), but no Group × Sex interaction (*F* = 0.1; *p* > 0.05). *significant *p* value.

Given that BMA-PACAPergic neurons modulate electrophysiological properties of dorsal mICC neurons, next, we wanted to test if, and how, this BMA (PACAP) to mICC (PAC1) pathway contributes to the learning and expression of fear behaviors. Our goal was to also determine whether gain of function in this pathway modulates any specific or all aspects of fear behaviors, so we tested the effects of optogenetic stimulation of BMA-mICC pathway on fear acquisition, generalization, recall, and extinction. We chose to look at all these aspects of fear in the same animals because mICCs have previously been shown to modulate fear extinction, but we wanted to interrogate whether this pathway has effects on other aspects of fear in addition to extinction in the same experimental preparation. Using the previously described intersectional approach in the Adcyap1-2A-Cre mice, we conducted an optogenetic gain-of-function experiment. We conducted bilateral *in vivo* optogenetic stimulation of PACAPergic fibers in the mICCs that emanate from the BMA and tested different aspects of contextual fear behavior, including acquisition, generalization, recall, and extinction. The behavioral procedure is shown in Figure 5*A*, *B*. These experiments were conducted in both males [*N* = 8 (ChR2); *N* = 9 (EYFP)] and females [*N* = 9 (ChR2); *N* = 7 (EYFP)]. Acquisition measures the ability of animals to learn the association between the context and shock, and an asymptotic level of conditional fear is graded in a manner that is proportional to shock intensity. ANOVA revealed a main effect of the Day (ANOVA; *F*_(4,145)_ = 66.503; *p* = 0.001) but no Sex × Group interaction (ANOVA; *F*_(1,145)_ = 0.833; *p* = 0.363) or Day × Sex × group interaction (ANOVA; *F*_(4,145)_ = 30.979; *p* = 0.980) in fear acquisition, indicating that the animals acquired/learned fear in a similar manner (Fig. 5*C*). Learned responses generalize to other contexts, and overgeneralization occurs in anxiety disorders reflecting fear in an inappropriate context. Moderate levels of freezing in this alternate context indicate that the mice exhibit overgeneralized fear. However, we found that the Sex × Group interaction in the generalization test was not significant, indicating that the animals did not differentially generalize fear to a novel context (Fig. 5*D*) (ANOVA; *F*_(1,29)_ = 0.594; *p* = 0.447).

The recall test was designed to measure the ability of animals to maintain a long-term fear memory of the context in which they acquired fear. ANOVA revealed a significant main effect of Group (ANOVA; *F*_(1,29)_ = 13.08; *p* = 0.001) in the recall test, such that the groups that received optogenetic stimulation of the PACAPergic neurons from BMA to mICCs showed a significantly reduced level of freezing compared with the EGFP controls (Fig. 5*E*). However, there was no significant Group × Sex interaction (ANOVA; *F*_(1,29)_ = 0.008; *p* = 0.993).

Extinction is the loss of expression of learned behavior with repeated exposure to the conditional stimulus (e.g., context) without the unconditional stimulus (e.g., shock). We found a main effect of Group (ANOVA; *F*_(1,145)_ = 20.128; *p* = 0.0001) and day (ANOVA; *F*_(4,145)_ = 26.907; *p* = 0.0001) during the extinction test, but no Group × Sex interaction (ANOVA; *F*_(1,145)_ = 0.1; *p* = 0.753) or Group × Sex interaction (ANOVA; *F*_(4,145)_ = 0.247; *p* = 0.911) (Fig. 5*F*). These results show that stimulation of PACAPergic pathway from BMA to mICCs alters specific aspects of fear behaviors and similarly between males and females. Specifically, our results indicate that the activation of PACAPergic pathway from BMA to mICCs decreases recall of fear, potentially indicating that these animals had a weaker memory of the context where they acquired fear behaviors. Although the recall was decreased, these animals also showed a propensity for enhanced extinction of fear in both males and females, indicating that activation of PACAPergic pathway from BMA to mICCs reduces fear of the context in which the context and shock associations were learned. While the freezing levels on extinction day 1 were higher than on the recall test, it is hard to know whether recall influenced extinction of fear.

### Deletion of PAC1 receptors from the mICCs enhances fear generalization and decreases fear extinction in males but not in females

**Figure 6.**
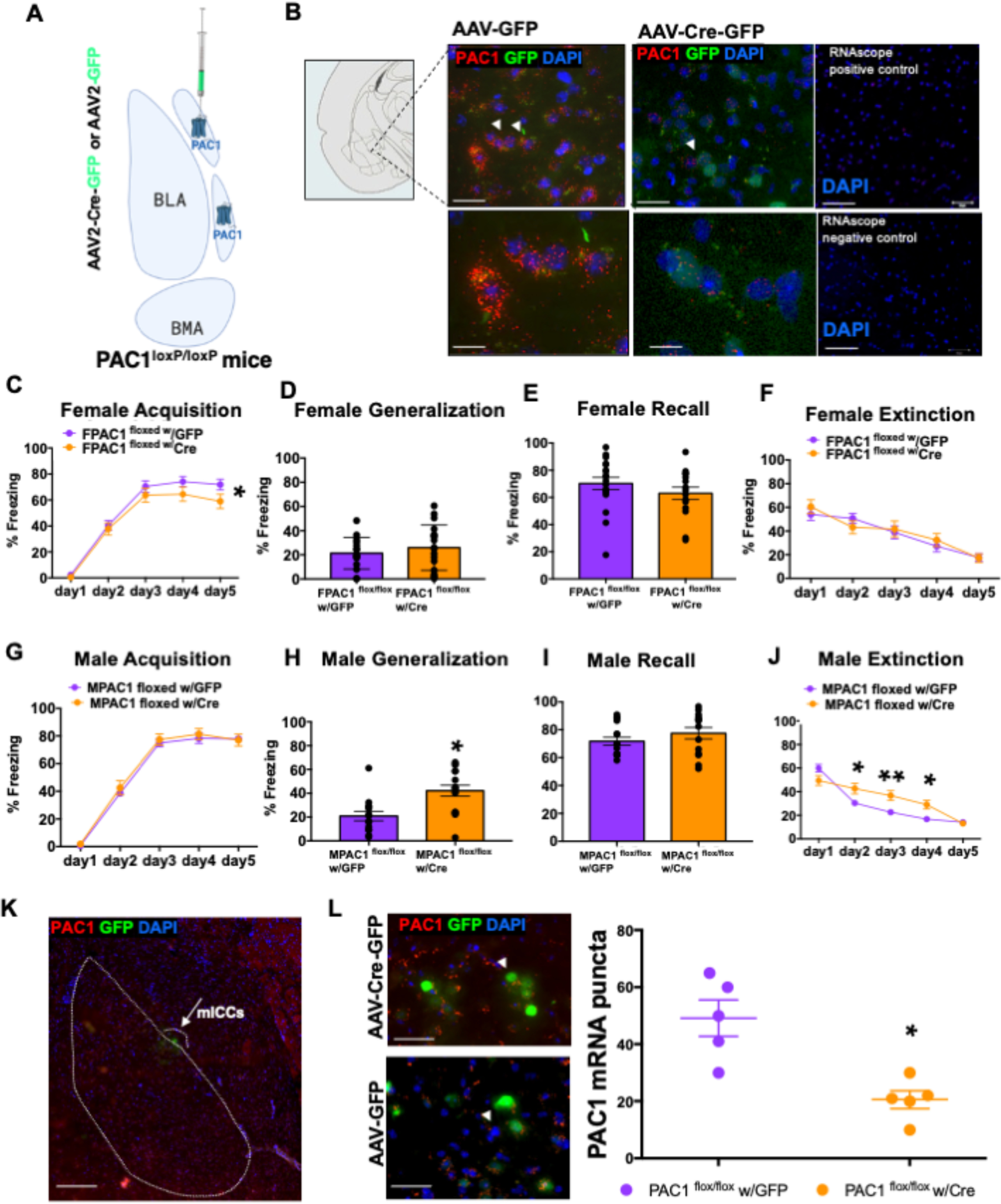
Deletion of PAC1 receptors from the mICCs of PAC1^loxp/loxp^ mice enhances fear generalization and decreases fear extinction in males but not in females. ***A***, Diagram represents the AAV2-Cre-GFP or control virus injections in the mICCs. Males (*N* = 14 [Cre]; *N* = 16 [GFP]) and females (*N* = 15 [Cre]; *N* = 18 [GFP]) were used in these experiments. ***B***, Example panels represent PAC1 expression levels in the mICCs from mice with AAV-GFP (left) and AAV-Cre-GFP (middle) using the RNAscope ISH technique. Right, Control sections with positive and negative controls. Arrows indicate PAC1 mRNA puncta. Scale bars, 25 µm. ***C***, ***G***, Graphs represent freezing during acquisition in males and females. ANOVA revealed a main effect of Day (*F* = 201.186, *p* = 0.0002), Sex (*F* = 10.586, *p* = 0.001), and a Sex × Group interaction (*F* = 4.757, *p* = 0.046) in acquisition. ***D***, ***H***, Graphs represent freezing during fear generalization test in males and females. There was a main effect of Group (*F* = 3.858, *p* = 0.05) and a Sex × Group interaction (*F* = 9.352, *p* = 0.003) on generalization test. *Post hoc* analysis showed that males with PAC1 deletion were significantly different from control (*p* < 0.05). ***E***, ***I***, Graphs represent fear recall test after deletion of PAC1 receptors from the mICCs in males and females. There was no effect of Sex or Group or Group × Sex interaction on the recall test (*F* = 2.463; *p* = 0.122). ***F***, ***J***, Graph represents freezing during extinction. There was a main effect of Day (*F* = 49.711, *p* = 0.0004), Sex (*F* = 12.513, *p* = 0.0002), and Day × Sex × Group interaction (*F* = 2.545, *p* = 0.04) in extinction. *Post hoc* analysis revealed that extinction rate in males with PAC1 deletion was significantly higher than controls on days 2-4 of extinction (*p* < 0.05). ***K***, Representative photomicrographs represent AAV-Cre-GFP injection into the mICCs. Arrow indicates GFP expression in mICCs. ***L***, Left, Representative photomicrographs show PAC1 mRNA puncta differences in mice that received AAV-Cre-GFP (top) or Control AAV-GFP (bottom) in the mICCs. Right, Graphs represent mean grain count of PAC1 mRNA in the mICCs with AAV-Cre-GFP (bottom) and control AAV-GFP (top). Mean grain count was significantly reduced after injection of AAV-Cre compared with AAV-GFP control (*p* < 0.05). Arrows indicate PAC1 mRNA puncta. Scale bars, 25 µm. *significant *p* value.

**Figure 7.**
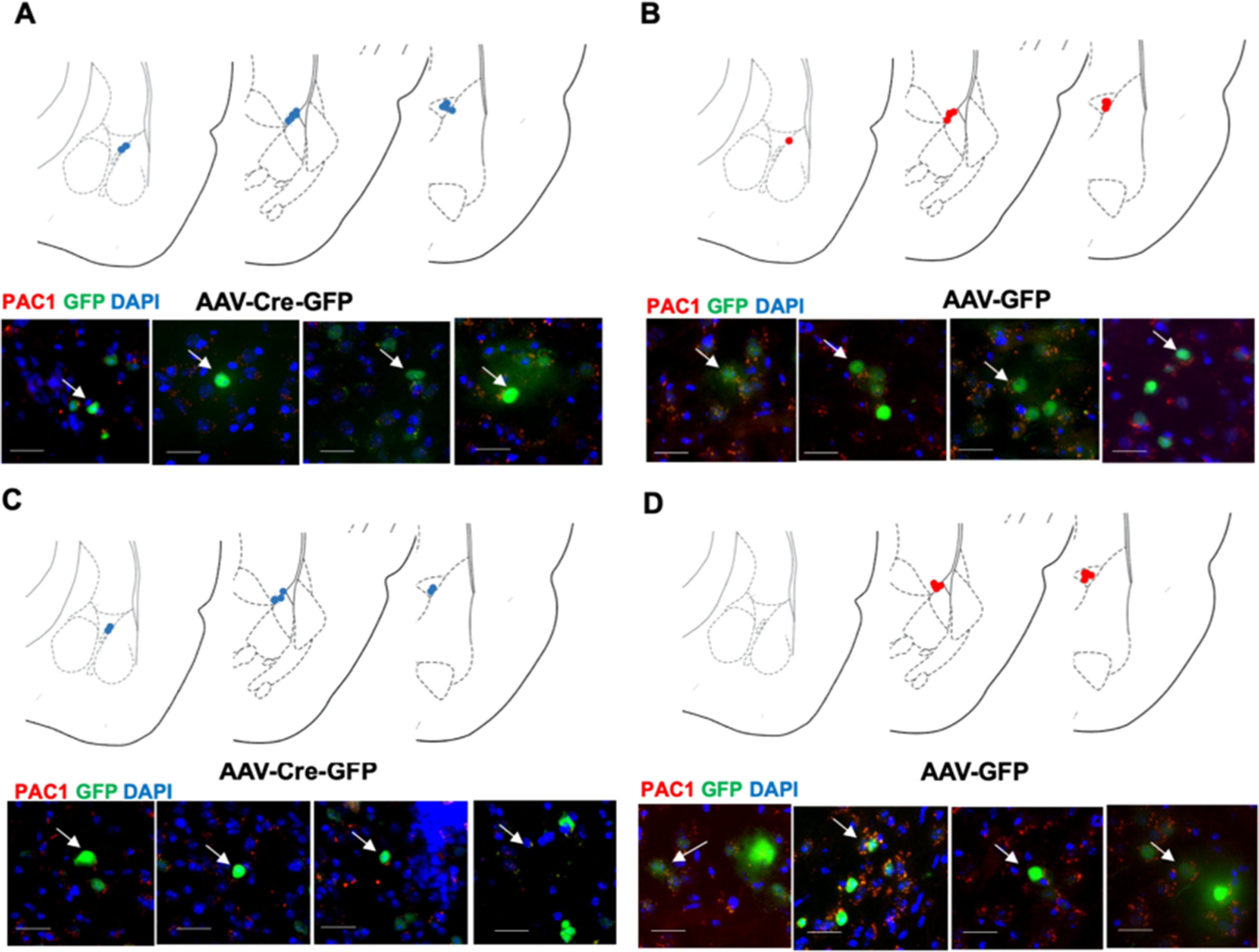
Composite example photomicrographs represent AAV-Cre-GFP-mediated deletion of PAC1 receptors in mICCs or control AAV-GFP without deletion across all groups. ***A***, Image panels and 4 example images represent viral targeting of mICCs in females with AAV-Cre-GFP-mediated PAC1 deletion. Arrows indicate mRNA puncta absence. Scale bars, 25 µm. ***B***, Image panels and 4 example images represent viral targeting of mICCs in females with AAV-GFP and no PAC1 deletion. PAC1 mRNA are shown in red puncta. Arrows indicate mRNA puncta. Scale bars, 25 µm. ***C***, Image panels and 4 example images represent viral targeting of mICCs in males with AAV-Cre-GFP-mediated PAC1 deletion. Arrows indicate mRNA puncta absence. Scale bars, 25 µm. ***D***, Image panels and 4 example images represent viral targeting of mICCs in males with AAV-GFP and no PAC1 deletion. PAC1 mRNA are shown in red puncta. Arrows indicate mRNA puncta. Scale bars, 25 µm.

Next, we wanted to determine the effects of loss of function of PAC1-containing cells in the mICCs on the same fear behaviors that were measured with optogenetic gain of function of the BMA-mICC PACAPergic pathway. Previous studies have shown that forebrain-specific deletion of PAC1 receptors leads to an impairment of contextual fear conditioning, but in these studies other aspects of contextual fear learning, such as generalization and extinction, were not examined (Otto et al., 2001). Also, mICCs have been shown to modulate fear extinction; but similar to the optogenetic stimulation experiments, we chose to specifically look at all phases of fear, including acquisition, generalization, recall, and extinction. This also allowed us to compare the loss-of-function experiment with PAC1 deletion in mICCs with the gain-of-function experiment with optogenetic stimulation of PACAPergic pathway to mICCs. ISH shows that PAC1 receptor mRNA expression is high in the mICCs compared with BLA/BMA or CN (Allen Brain Atlas). Thus, our loss-of-function experiment of PAC1 deletion in mICCs was designed to determine how a corresponding loss of mICCs PAC1 receptor gene expression affects the same set of fear behaviors as we tested with the optogenetic gain-of-function experiments (Fig. 6*A*,*B*). For this, we bilaterally injected the AAV2-hsyn-GFP-Cre or AAV2-hsyn-GFP as control into the dorsal mICCs of the mice with floxed PAC1 receptor gene. AAV2-hsyn-GFP-Cre allows Cre-mediated site-specific deletion of PAC1 receptors from the mICCs. Only the animals that had bilateral and specific viral expression localized in the mICCs were used in the behavioral analysis. We conducted testing in both males [*N* = 14 (Cre); *N* = 16 (GFP)] and females [*N* = 15 (Cre); *N* = 18 (GFP)]. ANOVA revealed a main effect of Day (ANOVA; *F*_(4,285)_ = 201.186, *p* = 0.0002), Sex (ANOVA; *F*_(1,285)_ = 10.586, *p* = 0.001), and a Sex × Group interaction (ANOVA; *F*_(1,285)_ = 4.757, *p* = 0.046) in acquisition (Fig. 6*C*,*G*). ANOVA revealed there was a main effect of Group (ANOVA; *F*_(1,59)_ = 3.858, *p* = 0.05) and a Sex × Group interaction (ANOVA; *F*_(1,59)_ = 9.352, *p* = 0.003) on the generalization test (Fig. 6*D*,*H*). *Post hoc* Bonferroni analysis showed that males with PAC1 deletion were significantly different from control (*p* < 0.05). ANOVA revealed no effect of Sex or Group or Group × Sex interaction on the recall test (Fig. 6*E*,*I*) (ANOVA; *F*_(1,59)_ = 2.463; *p* = 0.122). ANOVA revealed a main effect of Day (ANOVA; *F*_(1,295)_ = 49.711, *p* = 0.0004), Sex (ANOVA; *F*_(1,295)_ = 12.513, *p* = 0.0002), and Day × Sex × Group interaction (ANOVA; *F*_(4,295)_ = 2.545, *p* = 0.04) in extinction. *Post hoc* analysis revealed that extinction rate in males with PAC1 deletion was significantly lower than controls on days 2, 3 and 4 of extinction (*p* < 0.05) (Fig. 6*F* and Fig. 6*J*). Females showed decreased fear acquisition, but males showed enhanced fear generalization and decreased fear extinction. We verified that viral expression was localized in the mICCs with postmortem analysis using the RNAscope technique; PAC1 mRNA in the mICCs was significantly reduced after injection of AAV-Cre compared with AAV-GFP control (Figs. 6*K*,*L*, 7; paired *t* test; *t*_(4)_ = *p* < 0.05).

## Discussion

**Figure 8.**
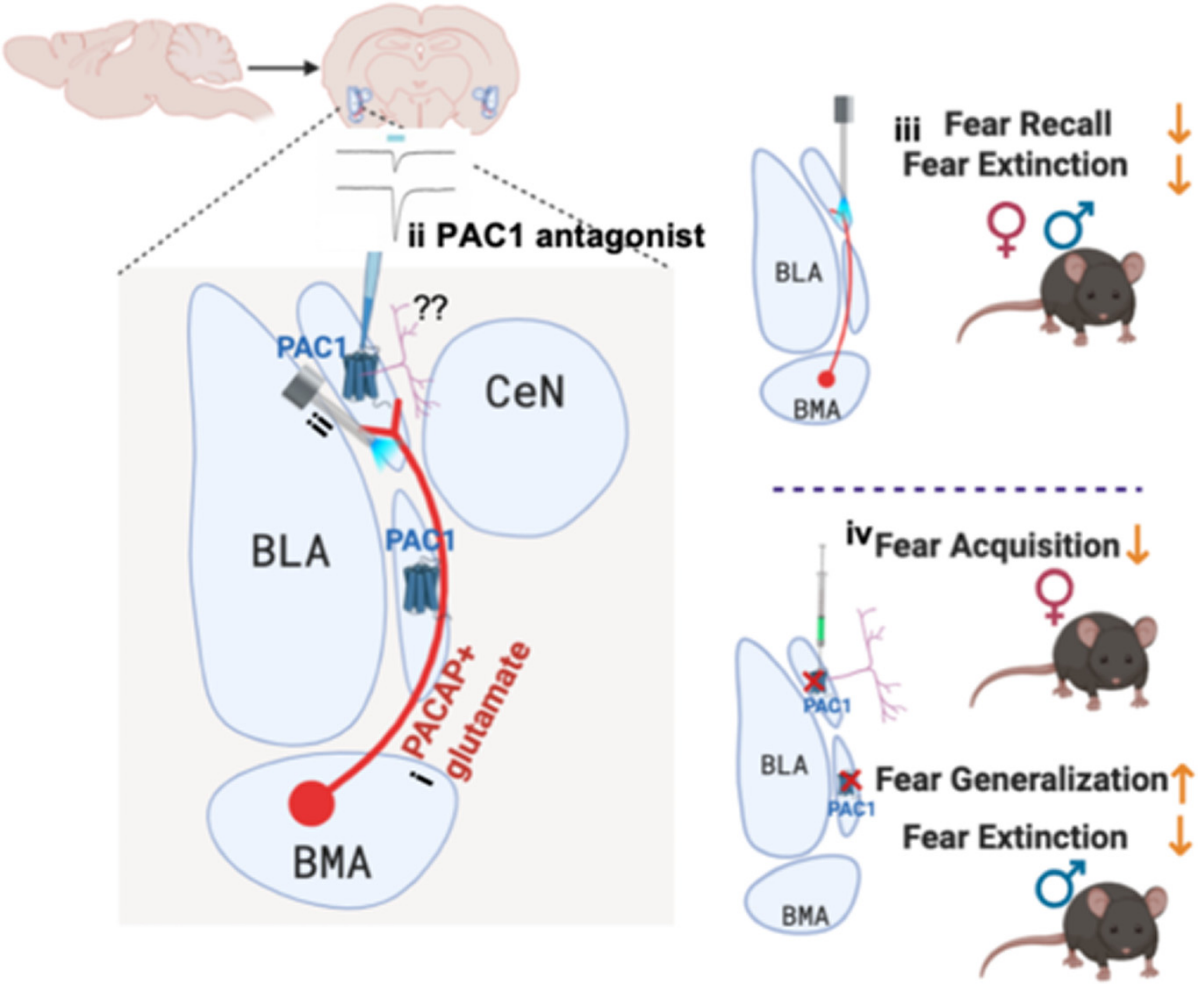
Working model of PACAP in the BMA and PAC1 in the mICCs in fear regulation through amygdala microcircuitry. A representative model of the amygdala microcircuitry containing PACAP neurons and PAC1 receptors in the BMA and mICCs, respectively. ***i***, BMA PACAPergic neurons are colocalized with glutamatergic neurons. ***ii***, Optogenetic activation of PACAPergic pathway from BMA to mICCs enhances excitatory postsynaptic potentials, which are further enhanced by application of PAC1 receptor antagonist. ***iii***, Stimulating PACAPergic terminals in the mICCs decreases fear recall and fear extinction. ***iv***, Deletion of PAC1 receptors from mICCs in females decreased fear acquisition, but deletion of these receptors from mICCs in males enhanced fear generalization and decreased fear extinction. This could occur through downstream projections of PAC1-expressing neurons in the mICCs.

We described a previously uncharacterized amygdala microcircuit containing the neuropeptide PACAP and PAC1 receptors in contextual fear regulation, whereby BMA PACAPergic neurons innervate the mICCs, which in turn express PAC1 receptors. BMA PACAP neurons regulate heterogeneous aspects of fear behaviors and electrophysiological properties of mICCs. PAC1-containing neurons in the mICCs, in turn, alter fear in a sexually dimorphic manner. Our results were supported by gain-of-function optogenetic, loss-of-function viral-mediated receptor deletion and electrophysiology using genetically modified mice (Working model: Fig. 8). While mICCs have previously been shown to be important for fear extinction (Likhtik et al., 2008), we uncovered that mICCs also play a role in influencing other components of fear memory, including acquisition, generalization, and recall.

We used a novel contextual fear conditioning procedure to assess fear acquisition, generalization, recall, and extinction together. Optogenetic stimulation of BMA-mICC PACAP reduced fear recall and extinction. Deletion of PAC1 receptors from mICCs led to increased fear generalization and decreased extinction in males and decreased acquisition in females. Using two complementary approaches, we found that the BMA sends dense PACAPergic projections to mICCs. Previous anatomic studies using the phytohemagglutinin lectin anterograde labeling method suggested that BMA projections to mICCs are sparse. While this method of labeling provides useful information about regional association, it is limited in providing information about transneuronal connections and genetically defined cell populations or defining functional synapses (Petrovich et al., 1996; Strick and Card, 2011).

Our electrophysiology results corroborated physiologically that mICCs are similar to medium spiny neurons, and functionally that these neurons show high excitability and input resistance (Millhouse, 1986; Royer et al., 2000). *Ex vivo* optogenetic stimulation of the PACAPergic BMA-mICC projections enhanced EPSCs with a short latency (10 ms) in mICC neurons. CNQX abolished the EPSCs, indicating that these responses were mediated by glutamatergic AMPARs. In a subset of neurons, TTX application suppressed EPSCs and TTX + 4-AP application increased EPSC amplitude, suggesting that the BMA-mICC pathway is monosynaptic. Application of PAC1 receptor antagonist (PACAP 6-38) concurrently with BMA-mICC optogenetic stimulation, surprisingly, further enhanced EPSCs. This suggests that PACAP is coreleased with glutamate, validated by our immunohistochemistry results represent VGLUT2 colocalized with PACAP. Blockade of PAC1R enhanced EPSC amplitude without changing paired-pulse ratio, arguing against the influence of PACAP 6-38 on presynaptic inputs. The molecular mechanism of how PAC1 blockade enhances EPSC amplitude remains to be precisely determined in future work. PAC1R are known to couple to K^+^ channels, and their activation can enhance K^+^ conductance in multiple cell types (Ichinose et al., 1998; Baron et al., 2001). One hypothesis is that the shunting inhibition promoted by basal tone of PACAP is relieved by application of PACAP 6-38, predicting EPSC enhancement from multiple sources outside of BMA. There were no sex differences in these measures. Our result suggests that BMA monosynaptic PACAPergic neurons drive behavioral effects by innervating a subset of mICC neurons.

*In vivo* optogenetic stimulation of the PACAP BMA-mICC pathway decreased contextual fear during the recall test in both males and females, indicating that the BMA-mICCs monosynaptic pathway may not be sexually dimorphic in and of itself. Following the recall test, we found extinction to be decreased over time. Given that recall itself was altered, we cannot conclude whether extinction changes are attributable to optogenetic manipulation during extinction or because of changes in recall. The differences in freezing during extinction emerged over the course of days, although the groups were at equivalent levels in the beginning, suggesting that PACAP BMA-mICC pathway modulates extinction, consistent with the known role of ICCs (Likhtik et al., 2008). Higher freezing on day 1 of extinction than recall suggests that extinction alterations may be independent of recall. While acquisition and generalization were not altered by our manipulation, it is not inconceivable that effects on fear encoding during acquisition influenced later recall and extinction. It is important to highlight that our main goal was to determine whether the BMA-PACAP circuit regulates any or all aspects of fear. Our findings indicate that BMA-mICC PACAP circuit diminishes specific aspects of learned fear expression rather than a general fear reduction, per se. Overall, our results lay a foundation for future experiments to precisely parse how this pathway affects specific aspects of fear (acquisition, generalization, etc.). For instance, does recall of generalization produce long-term plasticity affecting extinction? Other important future directions include determining mechanisms of optogenetic inhibition of the BMA-mICC (PACAP) pathway, interrogating if fear is changed by altered gating of BMA neurons or via direct influence on CN output neurons to promote behavior output. One limitation of our stimulation parameter where stimulation occurred throughout sessions instead of restricting to a phase of a session is the impact on future fear learning or unwanted effects. This was done to match the PAC1 deletion experiment, where the deletion was present throughout entire sessions.

PAC1 receptor deletion from mICCs slightly decreased fear acquisition, without altering generalization or extinction in females, somewhat modulating the asymptote of the learning curve. In males, PAC1 deletion from mICCs enhanced fear generalization and reduced extinction, indicating that these receptors regulate both fear generalization and extinction. Thus, in males, PAC1 receptors in BMA-mICC anatomic juncture may be necessary for regulating fear in an inappropriate context, whereas ablating them enhances fear. Reduced extinction indicates a failure to inhibit acquired fear when the PAC1 receptors are lacking. Overall, the results show that males and females show different fear expression when PAC1 receptors are absent in mICCs.

PAC1-containing mICC neurons project to various regions, in addition to the CN, to modulate distinct aspects of fear via these downstream projections. Our finding that sex differences in aspects of fear emerged only with the mICC receptor manipulation indicates that PAC1 containing mICC neurons receive innervation from other areas, in addition to the BMA, that drive behavioral output. While additional research is required, we hypothesize that PAC1-expressing mICC neurons and their downstream projections may be sexually dimorphic.

Our optogenetic stimulation and PAC1 deletion in the mICCs affected different aspects of fear. The optogenetic stimulation experiments were designed to modulate the upstream pathways of mICCs from the BMA, whereas the PAC1 deletion experiments modulate the downstream pathways. The mICCs are known to have a complicated morphology because of direct or indirect projections to the CN and could have different functional roles in fear regulation. Since more is known about the downstream projections of mICCs than the upstream projections to this region, our hypothesis is that the upstream pathways to mICCs are less heterogeneous, but the heterogeneous downstream projections affect multiple aspects of fear. Our conjecture fits with studies represent mICCs modulate distinct aspects of fear via specific neuropeptide/neurotransmitter microcircuits via projections to different structures (Amano et al., 2011; Busti et al., 2011; Duvarci and Pare, 2014). Also not in the scope of our paper, but subdivisions of the mICCs could also modulate different aspects of fear also via PACAP/PAC1.

We decided not to monitor cycle when comparing males and females because it is not possible to equate the stress from taking samples for cytology in females with males' experience. Therefore, we could not determine whether hormonal cycle played a role in the observed behavioral differences. Statistical analysis of heterogeneity of variance between males and females revealed similar variance for the male and female groups, suggesting that cycle may not play a major role in our observations. In future work, we will examine cycle effects directly by comparing only females at different stages of the cycle as that allows equivalent handling of all animals.

Amygdala microcircuitry is heterogeneous and complex in its morphology and function, and mICCs are major cell groups in this microcircuitry for processing specific aspects of fear-related information (Royer et al., 1999; Likhtik et al., 2008; Ehrlich et al., 2009; Palomares-Castillo et al., 2012; Pare and Duvarci, 2012). Our finding that PACAP and PAC1 influence specific fear properties suggests that PACAP/PAC1 is a critical neuropeptide system in the BMA-mICC amygdala node for differentially regulating sensory and associative information. Despite a complex cytoarchitecture, holistically, mICCs are plastic in their response to sensory information and modulate fear behaviors in a manner dependent on the incoming information (Amano et al., 2011; Huang et al., 2014; Asede et al., 2015).

While fear is a natural response that keeps organisms safe when faced with danger, fear dysregulation, as in PTSD, cripples an individual's ability to function. Traumatic stress results in enhanced acquisition of conditional fear that overgeneralizes to safe contexts or enhances recall and is less susceptible to extinction. We looked at all these aspects of fear in our study. PACAP and PAC1 have previously been shown to be important for fear regulation and high blood levels of PACAP, especially in females, and PAC1 methylation in a sex-independent manner is associated with PTSD (Ressler et al., 2011). Disentangling a microcircuit like BMA-mICCs in fear regulation via specific neuropeptide systems like PACAP/PAC1 provides better anatomic knowledge regarding substrates for targeted therapies for ameliorating symptoms in disorders like PTSD (Fendt and Fanselow, 1999).
